# Utilization of Torrefied and Non-Torrefied Short Rotation Willow in Wood–Plastic Composites

**DOI:** 10.3390/polym15193997

**Published:** 2023-10-05

**Authors:** Jaka Gašper Pečnik, Mariem Zouari, Matthew Schwarzkopf, David B. DeVallance

**Affiliations:** 1 InnoRenew CoE, Livade 6a, 6310 Izola, Slovenia; mariem.zouari@innorenew.eu (M.Z.); matthew.schwarzkopf@innorenew.eu (M.S.); 2 Faculty of Mathematics, Natural Sciences and Information Technologies, University of Primorska, Titov Trg 4, 6000 Koper, Slovenia; 3 College of Science and Technology, Commonwealth University of Pennsylvania, 401 North Fairview Street, Lock Haven, PA 17745, USA; ddevallance@lockhaven.edu; 4 Division of Forestry and Natural Resources, West Virginia University, Morgantown, WV 26506, USA

**Keywords:** torrefaction, wood–plastic composite, short rotation willow

## Abstract

The torrefaction process is widely used in the energy field, but the characteristics of the torrefied wood also have positive effects on the production of wood plastic composites. In this study, short-rotation shrub willow was torrefied at 225 and 300 °C and incorporated into polypropylene composites filled with changing levels of weight percent (wt%) of non-torrefied and torrefied (5, 15, 25, and 40 wt%) wood. Nine different formulations were extruded for mechanical, thermal, and water absorption properties. The tensile properties of composites were not affected by any level of torrefaction, while higher flexure properties were in favor of lower wt% of torrefied wood. The slowest rate of thermal degradation was confirmed for the highest wt% of torrefied wood with a torrefaction temperature of 300 °C. In contrast, the presence of torrefied wood in composites did not show a difference in crystallization or melting temperatures. The most noticeable contribution of torrefaction temperature and wt% was found for water-absorbing properties, where the higher torrefaction temperature and largest wt% of torrefied wood in the composite resulted in decreased water uptake.

## 1. Introduction

Woody crop biomass represents a significant source of renewable feedstock for bioenergy generation, bioproducts with a positive impact on soil and water conservation, recycled nutrients, and sequestered carbon [1]. Short-rotation wood species grown in plantations have higher potential than traditionally grown wood plantations due to shorter cultivation time intervals and high yields [2]. Agricultural perennial crops such as poplar and willow are considered energy crops grown for bioenergy production [3]. Short-rotation willows, such as Millbrook (*Salix purpurea* × *S. miyabeana*), with high growing yields, are often planted on marginal land from reclaimed mines or agricultural land to produce woody biomass. Willow stems grow rapidly and can be harvested every 3–4 years, up to 15 years. Biomass yield by dry stem mass in two growing seasons after coppice is estimated at around 9.3 T/year/hectare [4].

Willow biomass used in producing wood–plastic composites (WPC) was found to be an economically available source of natural fillers with improved and comparable mechanical performance of composites against classically used soft and hardwood fillers [5]. However, alternative pretreatments are key to adding value to fast-growing species grown primarily as energy crops to enhance the WPC material properties, specifically the hydrophobicity. WPCs are used in processed advanced materials in products for construction in the automotive industry, furniture applications, and consumer goods [6,7,8,9]. Wood is an environmentally friendly alternative to mineral fillers in the production of plastic composites [10]. Wood-based fillers have beneficial properties such as a good strength-to-weight ratio, low density, and easy integration into existing product lines [11]. Different types of wood fillers have been investigated to tailor the properties of WPC. Pudlik et al. [5] compared willow (*Salix viminalis*) filler to pine and beech fillers for WPC production and found comparable mechanical properties. Ayrilmis et al. [12] used fast-growing Pawlonia wood (*Paulownia elongata*) to produce WPC. They reported lower water absorption and thickness swelling properties against other fast-growing species such as Poplar spp. or Eucalyptus spp. The results were attributed to the lower capacity of bonding water, which depends on wood species. More recently, torrefied biomass has been gaining attention for non-energy applications. Tripathi et al. [13] indicated many applications where torrefied biomass is likely to outperform raw biomass and biochar. For example, the higher yield, as compared to biochar, and hydrophobic properties, as compared to raw biomass, make torrefied biomass a more attractive filler and additive in composites.

Kim et al. [14] observed small differences in tensile strength using several different wood species and pointed out that many wood species are appropriate for producing WPC. Another study [15] showed that selecting wood species for WPC material may improve its durability due to factors of water absorption and consequently create better conditions for decay growth [15]. Fabiyi et al. [16] found that water absorption in the case of wood species with lower diffusion coefficients also resulted in lower WPC water absorption due to differences in the chemical composition of the wood species. Bouafif et al. [17] reported that water absorption is also related to the particles size and contents in the composite material. Thermally modified wood is typically associated with being a more durable, dimensionally stable, and ecological alternative for construction products with a longer service life [18].

Moreover, studies related to WPC focused on using thermal modification wood to reduce water absorption, improve durability, and enhance the thermal resistance of WPC. For instance, Ayrilmis et al. [19] used cellulose fibers from fast-growing Eucalyptus camaldulensis treated at variable temperatures. They found that better water resistance for WPC panels was obtained when using thermally modified fibers. A review by Samaniego [20] also discussed applications of thermal treatments to improve wood-based composites, with the potential to apply the torrefaction process to enhance biomass properties. 

Torrefaction involves heating biomass between 200 °C and 300 °C in an inert atmosphere. The hemicelluloses are removed through this process, while the remaining material retains the original molecular structure of cellulose and lignin. Maintaining this structure provides the opportunity to utilize torrefied biomass in composite applications where cellulose and lignin have been shown to improve properties. During the grinding, the degraded hemicellulose matrix results in a more brittle material, which can impact the sphericity of particles and particle size [21]. By removing hemicellulose, torrefied wood can potentially improve WPC’s water-resistance properties. Recent torrefaction development has primarily focused on using biomass to improve caloric energy efficiency [22]. Besides the higher caloric value of torrefied biomass, the process enhances grindability, fungi decay, and water absorption. A few studies investigated using torrefied biomass as filler and reinforcement materials for polymers. 

Chiou et al. [23] found that adding torrefied biomass to polypropylene improved the distortion under heat and increased the glass transition temperature and tensile modulus of polypropylene. However, they found that the tensile strength decreased with increasing percentages of biomass. McCaffrey et al. [24] utilized torrefied almond shells when studying recycled polypropylene–polyethylene bio-composites. They reported that tensile and bending moduli could be increased by adding torrefied almond shells at high loading levels (up to 40%). They also reported that the particle size did not impact the mechanical properties. However, using smaller particles resulted in higher heat deflection temperature (HDT) values. Volfson et al. [25] found that torrefied pine filler in a 50% wood-to-polypropylene ratio improved the tensile strength by 38% and reduced the elongation at the break by 12%. They found a slight reduction in tensile strength when using torrefied birch filler. Vold et al. [26] investigated torrefied flax shives and sunflower hulls as fillers for polyamides. Their results suggested that the tensile elastic modulus of the polyamide was improved by adding torrefied flax filler up to 30%. Furthermore, the flexural and tensile modulus of the neat polyamide 6 (PA6) were found to increase, and the flexural and tensile strength decreased with increasing percentages (up to 30%) of torrefied sunflower hulls. They also reported a decrease in moisture uptake when torrefied fillers were added. This improvement in water absorption was also reported by Lu et al. [27], who found that the use of torrefied sweet sorghum slag fibers improved water absorption when used as fillers for high-density polyethylene (HDPE), as compared to non-torrefied sweet sorghum slag fibers. Berthet et al. [28] reported a 30% decrease in the water vapor permeability (WVP) of torrefied wheat straw fiber/poly(3-hydroxybutyrate-co-3-hydroxyvalerate) PHBV composites when using 20% of torrefied fibers. However, when loading levels were increased to 30%, fiber agglomeration occurred, and WVP increased. However, issues related to water absorption still exist when using willow as an additive for WPCs. On the other hand, torrefaction could be a useful pretreatment for short-rotation willow being used for composite-related applications. Research is necessary to determine how effective the torrefaction process, and therefore torrefied willow, is in improving the mechanical and physical properties of WPCs. Furthermore, while short-rotation willow is generally grown for energy-related applications, finding an alternative non-energy crop could provide growers with an additional value-added stream for their biomass feedstock. Given these opportunities, this study investigated non-torrefied and torrefied short-rotation shrub willow as a filler originating from low-value biomass and used to produce WPC. Combinations of polypropylene (PP), non-torrefied willow (NTW), and torrefied willow (TW) at varying weight percentages (wt%) were used to produce the WPCs. The TW was made using both 225 °C and 300 °C modification temperatures. The composites were evaluated under mechanical, thermal, and physical properties to assess the influence of filler particles as an alternative low-value filler to produce WPC. 

## 2. Materials and Methods

### 2.1. Materials 

Millbrook (*Salix purpurea × S. miyabeana*) short-rotation willow was used as a natural-based filler. The three-year-old willow was harvested at The Pennsylvania State University’s Rockview demonstration field site near State College, Pennsylvania, USA. The willow was ground without debarking into 4 mm particle size and air-dried to room equilibrium conditions (approximately 8% moisture content). Polypropylene (PP) pellets were supplied by PolyOne Corporation (Avon Lake, OH, USA), and a lubricant agent, Struktol TPW 113, a blend of complex modified fatty acid ester (Stow, OH, USA), was added to all formulations with a fixed percentage. 

### 2.2. Preparation and Characterization of the Filler

The woodchips were oven-dried to reach <5% moisture content for the torrefaction process. Two temperature levels of 225 °C and 300 °C were selected for the process. The torrefaction process occurred in the tube furnace under an inert atmosphere using N_2_ gas over a 30-min heating regime at a target temperature. The torrefied chips were ground in a Fritsch Pulverisette 25 grinder with a 1 mm mesh sieve size and stored in vacuum bags. The particle size distribution of dry torrefied wood (TW) and non-torrefied wood (NTW) flour was determined by laser scattering technology using a Horiba LA-960A2 analyzer (Horiba, Kyoto, Japan), and results for *D_10_*, *D_50_*, *D_90_*, and mean size are reported.

Fourier transform infrared spectra were obtained using an FTIR spectrometer (Bruker Optik, GmbH, Germany) connected to an ATR (attenuated total reflection) module. The spectra were collected over a wavelength range of 400 to 4000 cm^−1^ and a resolution of 4 cm^−1^. For each material (non-torrefied and torrefied at 225 °C and 300 °C), 64 scans were performed, and three repetitions were conducted to increase the accuracy of the results and reduce the effect of the atmospheric noise. Average spectra were collected using Opus software (https://www.bruker.com/en/products-and-solutions/infrared-and-raman/opus-spectroscopy-software/downloads.html, accessed on 28 September 2023). 

### 2.3. Blending and Production of WPC

In total, nine different samples of pellets were compounded using various mixing ratios of TW and NTW. At the same time, the amount of added polypropylene (PP) and lubricant remained constant at 56% and 4%, respectively. A sample of 0% TW was compounded without TW and served as a reference sample. For each torrefaction temperature level, four samples were compounded with variations in TW and NTW amounts (5, 15, 25, and 40% wt). Details about sample nomenclature and mixing composition are summarized in Table 1. 

The compounding of wood-PP pellets was conducted using the lab twin screw extruder (TSE 16TC) with four temperature zones heated at 185 °C, 190 °C, 200 °C, and 210 °C from the material feedthrough to the outlet die to ensure proper melting of the polymers. A constant feeding rate of 40 RPM was provided by an automatic single-screw feeder. For each formulation, the desired amount of different components (PP, filler, and lubricant) was mixed manually and then placed into the hopper.

During the extrusion process, the blend was cooled in a water bath located between the extruder nozzle and the pelletizer. The continuous line of extruded material was connected to the pelletizer with synchronized cutting and feeding speeds. Special care was taken to ensure that only well-blended and extruded material was pelletized and stored, disregarding the initial and end portions of the batch extrusion. This process limited the variability of extruded material in each blend and ensured the mixing process was as consistent as possible in the samples used for testing. Pellets were dried to remove excess water from the surface.

**Figure 1:**
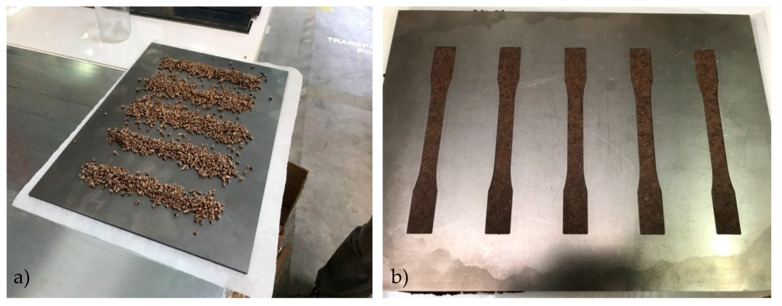
Manufacture of tensile specimens before (**a**) and after (**b**) the pressing process in a steel mold plate.

Steel molds were used to manufacture standardized testing specimens for tensile and three-point flexural tests according to ASTM D638 and ASTM D790, respectively. A 50 kN hydraulic hot press (LZT-UK-30-L, Langzauner, Lambrechten, Austria) equipped with water cooling was used to melt and compress compounded pellets into the molds for the final specimen shape. The steel plates were preheated to 200 °C in the hot press. Manufactured pellets were placed on the preheated molds and left for 10 min to soften. Waxed paper was inserted between steel plates to prevent samples from sticking. After softening the pellets, the heated plates were closed for 5 min at 200 °C. The cooling process followed the melting step by cooling the press plates to 80 °C. The mass of pellets per specimen volume was 11 g and 3 g for tensile and flexural mold, respectively, to reach a target density of approximately 1 g/cm^3^. Each pressing cycle generated five specimens of each shape. Figure 1 shows the process of manufacturing dog-bone specimens. 

### 2.4. Scanning Electron Microscopy

The morphology of the reference sample and the two samples with the largest TW levels at each temperature (40%TW-225 °C and 40%TW-300 °C) was evaluated by scanning electron microscopy (SEM). Samples were cut with the sharp blade, and images were collected using a scanning electron microscope (JEOL JSM-IT500, Oxford Instruments, Tokyo, Japan) operating at a low vacuum (70–80 Pa), a working distance of 10 mm, and an accelerated voltage of 15 kV.

### 2.5. Mechanical Properties

Tensile mechanical testing was performed on a universal testing machine (Zwick Roell, Ulm, Germany) with a 50 kN load cell equipped with hydraulic grips and extensometer arms. The testing speed was adjusted to 3 mm/min due to the brittle behavior of composites with a gauge length of 50 mm to determine the elongation of the specimen. The three-point bending flexural test was performed on a universal testing machine with a 1 kN load cell to ensure accurate measurements. The span was set to 53 mm, and the loading rate was 1.34 mm/min. Experiments were performed at room temperature (23 ± 2 °C) and 50 ± 10% relative humidity. For the rectangular flexural specimens, density was calculated before the test. Five and ten replicates were tested for tensile and flexural tests, respectively, and the average results were reported.

### 2.6. Water Absorption

After completing the flexural tests, ten rectangular specimens with approximate 24 mm × 13 mm dimensions were cut from the tested specimens and designated for water absorption tests. The specimens were oven-dried at 100 °C for 24 h, then weighed, and the initial mass (*m*_0_) was recorded. Specimens were then immersed in distilled water, and the mass after 24 h and 48 h (*m*_24_ and *m*_48_, respectively) was determined. Ten replicates were tested for each sample, and mean values were reported. 

### 2.7. Thermal Properties 

Thermogravimetric analyses (TGA) were conducted using a Discovery TGA (TGA 5500, TA Waters Instruments, New Castle, DE, USA). On average, 51 mg of specimen mass were placed on the aluminum pans and heated from room temperature up to 700 °C at a heating rate of 10 °C/min under nitrogen gas using a sample and balance purge flow of 25 mL/min and 10 mL/min, respectively. Data for *T_5%_*, *T_10%_*, and *T_max_* correspond to temperatures at which 5% and 10% weight loss were achieved, and temperatures at the maximum rate of specimen degradation (i.e., the highest points in the peaks appearing in the derivative curves) were extracted.

Differential scanning calorimetry (DSC) analysis was performed using a Discovery DSC 25 (TA Waters Instruments, New Castle, DE, USA). On average, 3.2 mg of sample in fine powder format were analyzed under a nitrogen gas atmosphere using the heat–cool–heat method as follows:

First heating cycle from 25 to 190 °C at 20 °C/min.
Cooling cycle from 190 to −30 °C at 20 °C/min.
Second heating cycle from −30 to 190 °C at 20 °C/min.

The first heating cycle was conducted to eliminate the thermal history of the composites. Data for crystallization temperature (*T_c_*), melting temperature (*T_m_*), and crystallization and melting enthalpies were determined from the cooling and second heating curves.

The degrees of crystallinity (*X_c_*) of the wood-PP-based composites were calculated using the following Equation (1):

(1) $$X_{c}=\frac{\Delta H_{m}}{w\times \Delta H_{m0}}\times 10$$

where *ΔH_m_* represents the samples’ melting enthalpies, *w* is the weight fraction of PP, and *ΔH_m_*_0_ is the melting enthalpy of 100% crystalline PP, which is equal to 207 J g^−1^ [29].

### 2.8. Statistical Evaluation

Data analysis was conducted in R (version 4.3.1) using RStudio (version 2023.06.0+421). The results of the measurements were reported as mean values with standard deviations (SD), including the number of analyzed specimens (No). An analysis of variance (ANOVA) was assessed to check for significant differences between samples that differ by TW loading levels and temperature levels. An ANOVA was conducted for each of the following outcomes: tensile modulus of elasticity, tensile strength, and flexural modulus of elasticity and flexural strength. When ANOVA showed a statistically significant result, post hoc pairwise comparisons were performed with Tukey’s HSD test for each of the above-mentioned outcomes (adjusted p values for multiple comparisons). The significance level was set at α = 0.05. 

## 3. Results and Discussion

This section may be divided by subheadings. It should provide a concise and precise description of the experimental results, their interpretation, and the experimental conclusions that can be drawn.

### 3.1. Particle Size of the Raw and Torrefied Wood Fillers

Size distributions of the non-torrefied wood (NTW) and torrefied wood (TW) reinforcement are presented in Table 2. Values for *D_10_*, *D_50_*, and *D_90_* correspond to the cumulative particle size percentiles and indicate the size below which 10%, 50%, and 90% of all particles are obtained, respectively.

The TW samples had a smaller particle size that ranged between <102 µm and 1140 µm and between <43 µm and 840 µm for fillers torrefied at 225 °C and 300 °C, respectively, compared to the NTW sample with a particle size between <163 µm and 1148 µm. The mean size decreased by 11% and 43% after torrefaction at 225 °C and 300 °C, respectively, indicating that the torrefaction process and temperature influenced the particles’ diameter. The decrease in particle size can be attributed to the shrinkage of the wood particles upon water evaporation during torrefaction. Moreover, the exposure to heat during torrefaction likely increased the grindability of the wood particles by provoking the decomposition of natural polymers, mainly hemicelluloses, that tend to degrade at temperatures ranging from 220 °C to 315 °C [21,30]. Therefore, pre-degraded particles were less resistant to the mechanical force during grinding after torrefaction. In this context, Moustafa et al. [31] reported that the average particle size of coffee grounds diminished gradually by 24% and 29% after torrefaction for 2 h at 250 °C and 270 °C, respectively. This was attributed to the shrinkage of particles under the heat effect. A similar trend with a decrease in particle size by increasing the modification temperature was also reported for temperatures at 160 and 170 °C for hydrothermally modified birch wood [32]. 

### 3.2. FTIR 

**Figure 2:**
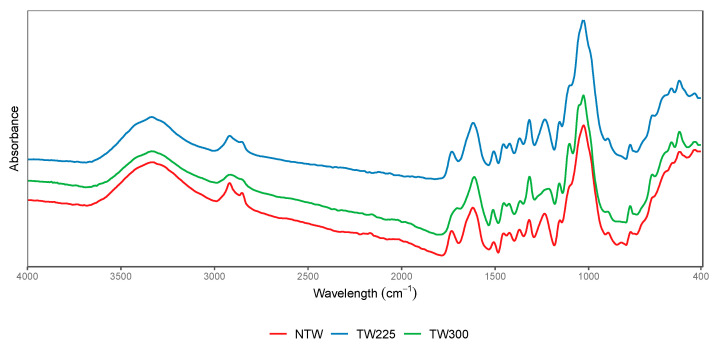
FTIR spectra of non-torrefied and torrefied wood biomasses.

The FTIR spectra of non-torrefied and torrefied wood particles are represented in Figure 2. The obtained spectra revealed some changes in the composition of the surface functional groups of the wood after the torrefaction treatment.

The broad peak at 3336 cm^−1^ was observed in all samples and was attributed to the occurrence of polar O-H stretching that corresponds to hydroxyl groups. These groups can correspond to water, alcohol, and carboxylic groups originating from natural polysaccharides and lignin [33]. The peak at 2916 cm^−1^ assigned to asymmetrical and symmetric -CH vibrations was detected in all samples. However, this peak was more visible in the non-torrefied wood material, while it tended to fade progressively in the materials torrefied at 225 °C and 300 °C. The reduction in -CH vibrations was likely associated with the decomposition of hemicellulose, given that they start the thermal degradation at a temperature ranging between 220 °C and 315 °C [30]. Similarly, a peak at 1730 cm^−1^ attributed to C=O functional groups from hemicelluloses was present in all samples. However, the peak intensity decreased after torrefaction at 300 °C [34].

Peaks at 1602 cm^−1^ and 1511 cm^−1^ were ascribed to C=C stretch vibrations and aromatic ring stretching from lignin. Similar peaks were observed previously in the FTIR spectra of Chinese fir and Eucalyptus globulus wood [33,34]. Peaks corresponding to lignin material remained almost constant after the torrefaction treatment, given that lignin has high thermal stability and its degradation occurs at a broad temperature range of 160 °C to 900 °C [30].

Peaks from 1000 to 1100 cm^−1^ attributed to C-O groups from cellulose were soundly prominent in all samples, suggesting that part of the cellulose in the wood materials remained intact after the torrefaction treatment, given that cellulose can degrade in a temperature range from 315 °C to 400 °C [30]. Prior research [35] reported that a similar peak vanished after treatment of the Arundo donax and olive stone biomass at high temperatures up to 600 °C.

### 3.3. SEM 

**Figure 3:**
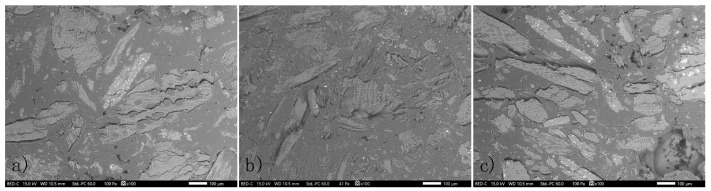
SEM images of the (**a**) reference sample (0%TW), (**b**) 40%TW-225 °C, and (**c**) 40%TW-300 °C at 100 µm magnification.

The distribution of wood particles and their interaction with the polymeric matrix were investigated by SEM at different magnifications. Images are presented in Figure 3, where the scale bar represents 100 µm. 

Wooden particles are randomly embedded in the polymer matrix, showing different slenderness. The wood particles were mostly long in shape, suggesting that they were horizontally oriented. However, often, the porous wooden structure can be observed. The difference between the samples (Figure 3) and the difference in the mean particle size (Table 2) is not clear in the images. The incorporation of wooden particles is also often loose from the matrix, indicating poor adhesion between the particles. It can also be observed that the wooden particles often collapse, and cell lumens are compressed together due to the high density of the material. The formation of aggregates was also observed in all samples, likely due to the poor distribution of the filler during the preparation process. Moreover, a void in the polymeric matrix was detected in Figure 3c, which can be attributed to the formation of air bubbles within the polymer during the blending process.

### 3.4. Mechanical Properties

Results from the tensile test are presented in Table 3. 

The values for modulus of elasticity ranged from 1293 MPa to 1476 MPa. Sample ME resulted in the highest modulus of elasticity with 1476 MPa, while the lowest MD showed 12% lower values. The post hoc pairwise comparisons between samples showed that only one pair had a statistically significant difference in average modulus of elasticity (ME-MD: 183 MPa, 95% CI: 8 to 358, *p* = 0.034). The results of the analyses are reported in Table S1 in the supplementary material. There is no clear explanation of these differences, but rather the variability in the material matrix composition. 

The results of tensile strength ranged between 9.9 MPa and 11.6 MPa (Table 3). Statistical analyses conducted with ANOVA on the tensile strength results revealed no statistically significant differences between samples. The results of post-hoc pairwise comparisons are presented in Table S2 in the supplementary material. These results indicate that *tw*% of TW loading and the temperature of torrefaction had no noticeable impact on the changes in tensile properties. Experimental results from three-point flexural tests are summarized in Table 4. 

Regarding flexural performance, the reference sample with 0%TW performed with the highest modulus of elasticity. Values for flexural strength showed that the strength values decreased with the increasing level of TW for the higher torrefaction temperature. Around 8% and 15% lower strengths were observed for the samples with the highest level of TW (ME, MI) at 225 °C and 300 °C against the reference, respectively. 

Results from the ANOVA suggested at least one group had a statistically significant difference for both flexural properties. In the case of the statistical analyses conducted for mean flexural modulus of elasticity, statistically significant differences were found for pairs MC-MA (−173 MPa, 95% CI: −319 to −27, *p* = 0.009), MF-MA (−174 MPa, 95% CI: −320 to −28, *p* = 0.008), MG-MA (−231 MPa, 95% CI: −377 to −85, *p* = 0.000), MH-MA (−200 MPa, 95% CI: −351 to −50, *p* = 0.002), MI-MA (−155 MPa, 95% CI: −301 to −9, *p* = 0.029), and MG-ME (−180 MPa, 95% CI: −362 to −34, *p* = 0.005). Apart from the MC-MA pair, the remaining statistically significant differences were found in samples with TW torrefied at 300 °C. In contrast, the MC sample had the lowest measured modulus of elasticity from TW at 225 °C. The complete results from post hoc analyses are presented in Table S3 in supplementary material. 

Post-hoc comparisons of flexural strength resulted in the following pairs being significantly different: MB-MA (−3.6 MPa, 95% CI: −6.5 to −0.7, *p* = 0.005), MC-MA (−3.1 MPa, 95% CI: −6.0 to −0.2, *p* = 0.026), MG-MA (−3.3 MPa, 95% CI: −6.2 to −0.4, *p* = 0.013), MH-MA (−4.6 MPa, 95% CI: −7.6 to −1.6, *p* = 0.000), MI-MA (−3.9 MPa, 95% CI: −6.8 to −1.0, *p* = 0.002), and pair MH-MF (−3.0 MPa, 95% CI: −6.0 to −0.0, *p* = 0.047). The remaining results from post hoc analyses are presented in Table S4 in supplementary material.

The flexural modulus of elasticity and strength performances of the reference sample containing NTW can be related to the higher average particle size (661 µm) compared to TW-225 °C and TW-300 °C (589 µm and 379 µm, respectively). With the higher torrefication temperature, a higher decrease in flexural properties was observed for 25 and 40% TW loadings. Torrefaction improves grindability and changes the chemical composition of the particles. This change can be attributed to different particle aspect ratios, such as the ratio between cellulose, lignin, and hemicellulose. In addition, smaller wood particles had a higher tendency to agglomerate, preventing uniform dispersion of the filler within the polymeric matrix, which can lead to lower flexibility of the material and poor adhesion of the matrix. Some of these phenomena were observed and described by other studies. Chiou et al. [23] injection molded extruded torrefied almond shells with wood chips and PP. They pointed out that the addition of particles to the matrix did not substantially impact the tensile modulus of elasticity but more on its strength, while with the increasing filler size, the modulus was reduced. However, mid and high particle size levels were subjected to 854 and 1545 um large particles in that prior study. Another study utilized torrefied almond shells (TAS) [24] with three different particle sizes and seven different loading levels in WPC. Increasing wt% of TAS increased the flexural modulus of elasticity, but samples with 50% TAS were already too brittle to evaluate flexural properties. The study found no effect of particle size on flexural properties. Regarding flexural strength, higher loadings of particles led to decreasing strength, while no effect on particle size was found. Chaudemanche et al. [36] evaluated the effect of particle size on extruded HDPE-WPC using three different size ranges and pointed out the importance of particle direction and aspect ratio. Flexural properties were better with larger particles. Also, in the tensile test, larger particles improved properties in the direction of sample extrusion. On the other hand, the finest particles resulted in better performance in the transverse direction. This result was attributed to smaller particles better aligning and compacting during processing into a composite. In our study, this might be true for flexural properties, while no changes due to particle size can be attributed to tensile properties. Pudlik et al. [5] also discussed the impact of chemical composition by comparing soft and hardwood species used in the HDPE-WPC. They emphasized that softwood species are more favorable for producing WPC due to their higher lignin and lower hemicellulose content. However, these properties result in higher strengths, lower flexibility, and poor internal bonding. In the study, the authors confirmed that a higher proportion of cellulose in the willow contributed to the greater strength of WPC. Due to the torrefaction process conducted in this study, these observations likely explain the reduction in flexural properties due to the lower mechanical properties of TW filler particles. Moreover, Patula pine biomass torrefied at 200 °C, 250 °C, and 300 °C showed that both lower torrefaction temperatures did not contribute to significant changes in chemical composition [37], which may also explain why more visible changes were also attributed to TW at 300 °C.

### 3.5. Water Absorption

**Figure 4:**
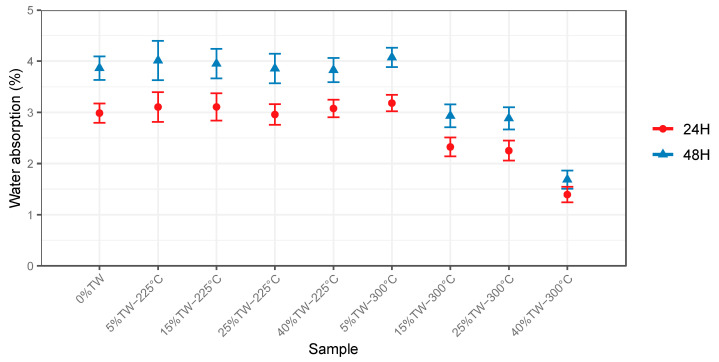
Change in water absorption variation in the wood-PP composites after 24 and 48 h.

Results of water absorption (WA) after 24 h and 48 h of immersion in water are presented in Figure 4. 

The water uptake of the reference sample (MA) reinforced with NTW (40 wt%) was about 3% and 3.9% after 24 and 48 h immersion, respectively (Figure 4). These findings agree with data reported by Ayrilmis et al. [19] for wood (50 wt%)-PP composites, where water uptake after one day was equal to 6.45%. Prior research [38] reported higher values for water absorption by wood (40 wt%)-PP material, which were about 4.5% and 6.3% after 24 h and 48 h, respectively. These differences in water absorption findings can be related to the different grades of the utilized PP matrix (ratio of crystalline and amorphous regions) or the dimensions of the tested material.

The WA remained steady after incorporating 5, 15, 25, and 40 wt% of wood treated at 225 °C and 5 wt% treated at 300 °C. However, it started to decrease proportionally when the TW-300 °C load was further increased to 15, 25, and 40 wt%, and the decrease in water uptake after 24 h was 2.3, 2.2, and 1.4%, respectively, compared to the reference sample (MA). These findings show that a higher torrefaction temperature (i.e., 300 °C) had a greater effect on the hygroscopic behaviour of the composites. The increase in torrefaction temperature likely contributed to the degradation of most of the present hemicelluloses, which are characterized by their hydrophilic character. 

Prior research [39] investigated the relationship between torrefaction treatment and the moisture uptake of woody biomass. They utilized softwood (*Fir*, *Abies pectinates*) samples torrefied at variable temperatures (from 200 °C to 230 °C for 1000 min). They reported that the equilibrium moisture content of torrefied wood decreased by 34 and 58% with the increase in torrefaction temperature from 200 to 230 °C, respectively. The authors explained that the hydroxyl groups present in the biomass, mainly hemicelluloses and amorphous regions of celluloses, are responsible for attracting and retaining water molecules via hydrogen bonding. These groups were hydrolyzed during exposure to heat, which increased the hydrophobicity of the material. Similarly, pressed wood plastic panels made of an equal ratio (50:50) of thermally treated wood fibers (*Eucalyptus camaldulensis*) and PP matrix were manufactured by Ayrilmis et al. [19], and the hygroscopic features were explored. They found that the composites water absorption (in 24 h) significantly decreased with increasing the treatment temperature from 120 °C to 180 °C (40 min) by 33.7%, which was linked to the degradation of hydrophilic hemicelluloses.

### 3.6. Thermal Properties 

#### 3.6.1. Thermogravimetric Analysis 

**Figure 5:**
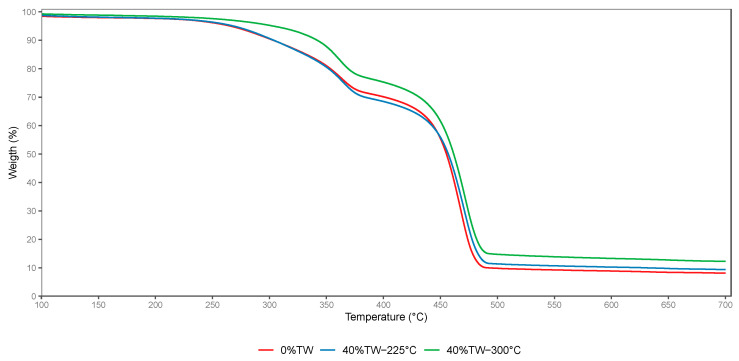
TGA thermograms of 0%TW, 40%TW-225 °C, and 40%TW-300 °C.

The TGA mass loss plots obtained for NTW and TW-reinforced PP composites were very similar, and the curves overlapped. Therefore, only curves from the reference sample (0%TW), 40%TW-225 °C, and 40%TW-300 °C were selected and represented in Figure 5. The degradation temperature points *T_5_*_%_, *T_10_*_%_, and *T_max_* corresponding to 5%, 10%, and maximum weight loss, respectively, are summarized in Table 5.

The thermal decomposition started earlier for the 0%TW (reference sample MA) compared to the reinforced samples. The incorporation of TW reinforcement had a favorable effect on the material’s thermal resistance. Thermal degradation was delayed, especially when TW-300 °C was utilized. When 5% of TW-225 °C was incorporated, *T_5%_* and *T_10%_* decreased slightly, whereas *T_max_* remained steady. Thus, no remarkable changes were observed in the thermal degradation compared to the reference sample. However, when the loading of TW-225 °C was further increased, *T_5%_*, *T_10%_*, and *T_max_* increased. The highest increment in *T_5%_* and *T_10%_* was 6.4 °C and 5.3 °C, respectively, for the sample containing 25% of TW (MD), while *T_max_* remained almost the same compared to the reference sample. Up to 25% of TW, the increase in TW loading had a negative impact on the thermal stability, and a decrease in the degradation points was noted (Table 5). When TW-300 °C was used as reinforcement, *T_5%_*, *T_10%_*, and *T_max_* increased gradually with the filler loading up to 40%, with a slight difference observed between samples loaded with 15% and 25%. The highest thermal stability was exhibited by sample 40% TW-300 °C (MI) with an elevation in *T_5%_*, *T_10%_*, and *T_max_* of 37.6 °C, 38.5 °C, and 4.4 °C, respectively, compared to the reference sample. The final residue percentage increased with the increase in TW percentage from 5% to 40%, and the increment was more significant in the case of filler treated at 300 °C. The final residue at 700 °C increased by 16% and 52% for samples with 40%TW treated at 225 °C (ME) and 300 °C (MI) compared to the reference sample. These findings suggested that utilizing TW as a reinforcement for PP, particularly TW-300 °C, could improve the thermal stability of wood–polymer composites at high temperatures. The enhancement in thermal resistance can be attributed to torrefied wood’s higher stability than raw wood, owing to the pre-degradation of hemicelluloses during the treatment. Hemicellulose is known as the most thermolabile natural polymer, given that it starts to degrade at earlier stages (from 220 °C to 315 °C [30]) compared to other wood components (i.e., cellulose and lignin). TW filler likely contained reduced hemicellulose content, which improved the thermal heat resistance and led to the generation of more residue at the end of the heating cycle. Barajas et al. [40] observed similar trends when they studied the thermal properties of PLA composites reinforced with raw and torrefied (250 °C) coffee husk flour (CHF). They reported that *T_5%_* and *T_max_* of PLA-20% torrefied CHF increased by 23 °C and 4 °C, respectively, compared to PLA-20% raw CHF. The authors explained that the torrefaction led to removing the less thermally stable components, specifically hemicellulose and cellulose. However, they found that a further increase in the torrefied CHF loading (up to 50%) negatively affected the composites thermal stability, attributed to the potential occurrence of metals and water in the filler powder that catalyzed and favored the thermal degradation. Moreover, they found that [40] observed similar trends when they studied the thermal properties of PLA composites reinforced with raw and torrefied (250 °C) coffee husk flour. They reported that *T_5%_* and *T_max_* decreased gradually from 322.4 ± 1.7 °C to 301.1 ± 1.3 °C and from 354.7 ± 2.2 °C to 341.4 ± 1.6 °C, respectively, when torrefied coffee husk loadings were increased from 20% to 50% wt%. However, they recorded a continuous increase in residual mass from 5.6 ± 0.5 to 10.9 ± 0.8% (from 20 to 50 wt% loading). They also reported that within the same filler loading (20 wt%), *T_5%_* and *T_max_* of composites with torrefied filler increased by 23.5 °C and 3.6 °C, respectively, and final residue at 700 °C increased continuously with the increase in torrefied CHF content, which was justified by the ability of torrefied material to generate carbon-rich residue. Similarly, prior research [31] stated that the thermal resistance of polybutylene adipate terephthalate (PBAT) composites was enhanced when the coffee ground was torrefied at 270 °C when incorporated, compared to composites containing raw or coffee ground torrefied at a lower temperature (250 °C). The findings were attributed to the hydrolysis of less heat-stable compounds during the torrefaction, which increased with the severity of the torrefaction temperature (i.e., from 250 to 270 °C).

#### 3.6.2. Differential Scanning Calorimetry

DSC-derived heating and cooling isotherms for the reinforced PP composites were considered to understand the effect of the torrefied filler’s incorporation on the PP polymeric matrix’s crystallization and melting behaviors. The crystallization temperature (*T_c_*), crystallization enthalpy (*ΔH_c_*), melting temperature (*T_m_*), melting enthalpy (*ΔH_m_*), and crystallinity degree (*X_c_*) of all samples are listed in Table 6.

The crystallization temperature remained steady at 5% and 15% TW loadings, regardless of the torrefaction temperatures. As the amount of TW-225 °C increased to 25%, *T_c_* increased by 4 °C compared to the reference sample. However, the increment in *T_c_* was negligible in the case of 25% TW-300 °C (MH).

When TW levels were further increased to 40%, *T_c_* decreased by 3.5 °C and 1.6 °C in the case of 250 °C and 300 °C torrefied fillers, respectively, compared to the corresponding 25% samples. The *T_c_* values of composites reinforced with TW-250 °C wood filler were higher than those reinforced with TW-300 °C within the same loading levels (Table 6). This result was attributed to the differences in particle size that affected the dispersion of the wood particles within the matrix. TW-225 °C had a larger average particle size (589 µm) compared to TW-300 °C (379 µm). Bigger particles likely prevented the formation of crystals by creating inter-spaces between the PP chains, delaying the *T_c_* from reaching higher values. The degree of crystallization decreased as the amount of TW increased, from 5% to 40%, regardless of the torrefaction temperature. Therefore, with the addition of higher TW loadings, the formation of crystals within the polymeric matrix was harder, which means that the wood particles likely limited the nucleation of the materials by lowering the mobility of PP molecular chains.

*T_m_* values remained almost constant for all samples except for composites containing 25% and 40% of TW-300 °C (MH and MI), where a slight decrease was observed (Table 6). Chiou et al. [41] obtained similar results, incorporating variable loadings (5, 12.5, and 20 wt%) of almond shell torrefied at 280 °C and 300 °C and wood chips torrefied at 280 °C in a PP matrix. They reported that melting temperature values for all the specimens ranged between 166 °C and 167 °C, comparable to the neat PP (167 °C). They concluded that the filler did not significantly influence the melting behaviors of the PP chains. Moustafa et al. [31] reported different findings when they studied the different properties of PBAT composites reinforced with raw and torrefied coffee ground at variable loadings (10, 20, and 30%). They found that torrefied filler had a favorable effect on the thermal resistance of PBAT composites. The *T_m_* of PBAT filled with untreated coffee ground ranged between 96.7 °C and 97.4 °C, whereas samples filled with coffee ground torrefied at 250 °C had higher *T_m_* values that ranged between 109.8 °C and 115.1 °C. However, using coffee ground torrefied at higher temperatures (270 °C) reduced the *T_m_*, and values ranged between 95.8 and 105.6 °C.

## 4. Conclusions

Wood plastic composites were prepared from polypropylene reinforced with torrefied and non-torrefied short rotation willows via extrusion and molding processes. The morphology, mechanical, thermal, and water absorption properties of the materials were examined. The mean particle size of wood fillers decreased by 11% and 43% after torrefaction at 225 °C and 300 °C, respectively, suggesting that the torrefied particles had better grindability. Observation of SEM images revealed the random distribution of filler particles and the occurrence of voids within the polymeric matrix. The flexural strength decreased with the increase in torrefied wood particles, and all samples’ flexural modulus of elasticity was lower than the reference sample. Changes in flexural behaviors may be attributed to differences in wood particle size and distribution within the polymeric matrix. However, the tensile strength did not change significantly. Samples containing particles torrefied at 300 °C exhibited higher water resistance than other samples. The TGA results showed that the addition of torrefied wood particles delayed the thermal degradation of the composites. The thermolabile components in the wood were partially removed by torrefaction, which enhanced the thermal stability of the composites. The crystallization degree decreased with increased torrefied wood loadings while the melting temperatures remained almost steady.

Torrefied short-rotation willow is a good candidate as a low-value-added filler in polypropylene and wood–polypropylene composites. The optimization of the filler’s loadings is crucial to achieving optimal performance for the final composites.

## Figures and Tables

**Table 1:** Formulation of blending mixtures with sample name, the ratio of non-torrefied and torrefied wood, and torrefaction temperature.

Sample	Sample Description	NTW	TW	Torrefaction Temperature
MA	0%TW	40%	0%	No torrefaction
MB	5%TW-225 °C	35%	5%	225 °C
MC	15%TW-225 °C	25%	15%	
MD	25%TW-225 °C	15%	25%	
ME	40%TW-225 °C	0%	40%	
MF	5%TW-300 °C	35%	5%	300 °C
MG	15%TW-300 °C	25%	15%	
MH	25%TW-300 °C	15%	25%	
MI	40%TW-300 °C	0%	40%	

**Table 2:** Representative particle size diameters of the woody fillers.

Cumulative Particle Size Distribution	Particle Size (µm)		
	NTW	TW 225 °C	TW 300 °C
D_10_	163	102	43
D_50_	639	530	290
D_90_	1148	1140	840
Mean	661	589	379

**Table 3:** Mean tensile strength and modulus of elasticity with standard deviation (SD) and number of recorded specimens (No.).

Sample	Modulus of Elasticity		Tensile Strength		
	(MPa)	SD	(MPa)	SD	No.
MA	1336	84	10.8	0.7	4
MB	1417	86	11.6	1.1	5
MC	1426	47	11.3	0.6	5
MD	1293	80	9.9	1.0	5
ME	1476	48	11.0	0.2	5
MF	1431	118	11.6	0.8	5
MG	1361	93	10.5	1.8	5
MH	1396	26	10.5	0.5	5
MI	1398	119	11.0	0.7	5

**Table 4:** Mean values for flexural modulus of elasticity, strength, density with standard deviation (SD), and number of specimens (No).

Sample	Modulus of Elasticity		Flexural Strength			Density		
	(MPa)	SD	(MPa)	SD	No.	(kg/m^3^)	SD	No.
MA	1777	123	26.6	2.2	10	973.8	32.5	10
MB	1682	75	23.0	1.6	10	975.3	9.3	10
MC	1604	82	23.5	1.8	10	992.1	8.9	10
MD	1663	122	24.0	3.2	10	981.7	7.5	10
ME	1726	97	24.5	1.6	10	997.6	9.0	10
MF	1603	81	25.0	1.4	10	984.6	8.3	10
MG	1546	36	23.3	1.6	10	980.5	12.5	10
MH	1577	175	22.0	2.8	9	984.6	7.4	10
MI	1622	82	22.7	1.4	10	979.3	7.6	10

**Table 5:** Thermal degradation points and final residue of TW and NTW-reinforced PP.

Sample	Degradation Temperature (°C)			Final Residue at 700 °C (%)
	*T_5%_*	*T_10%_*	*T_max_*	
MA	265.8	303.4	468.2	8.1
MB	263.2	302.2	468.3	8.9
MC	271.6	307.8	466.8	8.6
MD	272.3	308.7	468.6	8.5
ME	268.5	303.9	471.0	9.4
MF	271.9	309.4	471.0	9.2
MG	281.9	323.5	470.9	9.7
MH	280.0	322.6	469.4	10.9
MI	303.4	342.0	472.6	12.3

**Table 6:** Thermal transitions of the wood-PP-based composites.

Sample	*T_c_* (°C)	*ΔH_c_* (J g^−1^)	*T_m_* (°C)	*ΔH_m_* (J g^−1^)	*X_c_* (%)
MA	112.7	48.0	160.5	40.3	32
MB	112.6	64.7	160.9	51.6	42
MC	113.2	52.5	160.4	40.5	33
MD	116.7	56.8	160.6	35.7	29
ME	113.2	44.8	160.1	31.2	25
MF	112.9	62.0	160.6	41.2	33
MG	112.4	44.3	160.8	30.7	25
MH	113.1	34.2	159.2	25.5	21
MI	111.4	48.3	159.6	34.2	28

## Data Availability

The data presented in this study are available on request from the corresponding author.

## Supplementary Materials

The following supporting information can be downloaded at: https://www.mdpi.com/article/10.3390/polym15193997/s1. Table S1: Results of the Tukey HSD comparisons between pairs for tensile modulus of elasticity with difference between the pairs, 95% confident interval and *p*-value. Table S2: Results of the Tukey HSD comparisons between pairs for tensile strength with difference between the pairs, 95% confident interval and *p*-value. Table S3: Results of the Tukey HSD comparisons between pairs for Flexural modulus of elasticity with difference between the pairs, 95% confident interval and *p*-value. Table S4: Results of the Tukey HSD comparisons between pairs for Flexural strength with difference between the pairs, 95% confident interval and *p*-value.
