# Supplementary material for: Utilization of Torrefied and Non-Torrefied Short Rotation Willow in Wood–Plastic Composites
Source: Polymers (Basel). 2023 Oct 5;15(19):3997. doi: 10.3390/polym15193997 (PMC10575387; doi:10.3390/polym15193997)
Supplement: Supplementary file 1 [file polymers-15-03997-s001.zip › polymers-2616202-supplementary.pdf]

Table S1: Results of the Tukey HSD comparisons between pairs for tensile modulus of elasticity with difference between the pairs, 95 % confident interval and p-value.

**Tensile modulus of elasticity**				
Sample	*difference*	*95 % confidence interval*		*p adjusted*
MB-MA	81	-104	267	0.869
MC-MA	90	-95	275	0.794
MD-MA	-43	-228	142	0.997
ME-MA	140	-45	325	0.268
MF-MA	95	-90	280	0.745
MG-MA	25	-160	211	1.000
MH-MA	60	-125	245	0.974
MI-MA	62	-123	247	0.969
MC-MB	9	-166	183	1.000
MD-MB	-124	-299	50	0.340
ME-MB	59	-116	233	0.969
MF-MB	14	-161	188	1.000
MG-MB	-56	-231	119	0.976
MH-MB	-21	-196	153	1.000
MI-MB	-19	-194	155	1.000
MD-MC	-133	-308	41	0.258
ME-MC	50	-125	224	0.989
MF-MC	5	-170	179	1.000
MG-MC	-65	-239	110	0.945
MH-MC	-30	-204	145	1.000
MI-MC	-28	-202	147	1.000
**ME-MD**	**183**	**8**	**357**	**0.034**
MF-MD	138	-37	313	0.219
MG-MD	68	-106	243	0.927
MH-MD	103	-71	278	0.585
MI-MD	105	-69	280	0.560
MF-ME	-45	-220	130	0.994
MG-ME	-115	-289	60	0.447
MH-ME	-80	-254	95	0.844
MI-ME	-78	-252	97	0.861
MG-MF	-70	-244	105	0.919
MH-MF	-35	-209	140	0.999
MI-MF	-33	-207	142	0.999
MH-MG	35	-140	209	0.999
MI-MG	37	-138	211	0.999
MI-MH	2	-173	177	1.000

Table S2: Results of the Tukey HSD comparisons between pairs for tensile strength with difference between the pairs, 95 % confident interval and p-value.

Tensile strength

Sample	*difference*	*95 % confidence interval*		*p adjusted*
MB-MA	0.8	-1.2	2.8	0.929
MC-MA	0.5	-1.6	2.5	0.996
MD-MA	-0.9	-2.9	1.1	0.873
ME-MA	0.3	-1.8	2.3	1.000
MF-MA	0.8	-1.2	2.9	0.913
MG-MA	-0.2	-2.3	1.8	1.000
MH-MA	-0.2	-2.3	1.8	1.000
MI-MA	0.2	-1.8	2.2	1.000
MC-MB	-0.3	-2.2	1.6	1.000
MD-MB	-1.7	-3.6	0.2	0.125
ME-MB	-0.5	-2.4	1.4	0.992
MF-MB	0.0	-1.9	2.0	1.000
MG-MB	-1.0	-2.9	0.9	0.706
MH-MB	-1.0	-2.9	0.9	0.707
MI-MB	-0.6	-2.5	1.3	0.982
MD-MC	-1.4	-3.3	0.5	0.332
ME-MC	-0.2	-2.1	1.7	1.000
MF-MC	0.3	-1.6	2.3	1.000
MG-MC	-0.7	-2.6	1.2	0.943
MH-MC	-0.7	-2.6	1.2	0.943
MI-MC	-0.3	-2.2	1.6	1.000
ME-MD	1.2	-0.8	3.1	0.552
MF-MD	1.7	-0.2	3.6	0.111
MG-MD	0.7	-1.3	2.6	0.965
MH-MD	0.7	-1.3	2.6	0.964
MI-MD	1.1	-0.8	3.0	0.630
MF-ME	0.6	-1.4	2.5	0.988
MG-ME	-0.5	-2.4	1.4	0.993
MH-ME	-0.5	-2.4	1.4	0.993
MI-ME	-0.1	-2.0	1.9	1.000
MG-MF	-1.1	-3.0	0.9	0.672
MH-MF	-1.1	-3.0	0.9	0.673
MI-MF	-0.6	-2.5	1.3	0.975
MH-MG	0.0	-1.9	1.9	1.000
MI-MG	0.4	-1.5	2.4	0.998
MI-MH	0.4	-1.5	2.4	0.998

Table S3: Results of the Tukey HSD comparisons between pairs for Flexural modulus of elasticity with difference between the pairs, 95 % confident interval and p-value.

**Flexural modulus of elasticity**				
Sample	*difference*	*95 % confidence interval*		*p adjusted*
MB-MA	-95	-241	51	0.500
**MC-MA**	**-173**	**-319**	**-27**	**0.009**
MD-MA	-114	-260	32	0.254
ME-MA	-51	-197	95	0.971
**MF-MA**	**-174**	**-320**	**-28**	**0.008**
**MG-MA**	**-231**	**-377**	**-85**	**0.000**
**MH-MA**	**-200**	**-351**	**-50**	**0.002**
**MI-MA**	**-155**	**-301**	**-9**	**0.029**
MC-MB	-78	-224	68	0.744
MD-MB	-19	-165	127	1.000
ME-MB	44	-102	190	0.988
MF-MB	-79	-225	67	0.731
MG-MB	-136	-282	10	0.089
MH-MB	-105	-256	45	0.393
MI-MB	-60	-206	86	0.926
MD-MC	59	-87	205	0.932
ME-MC	122	-24	268	0.179
MF-MC	-1	-147	145	1.000
MG-MC	-58	-204	88	0.938
MH-MC	-27	-178	123	1.000
MI-MC	18	-128	164	1.000
ME-MD	63	-83	209	0.904
MF-MD	-60	-206	86	0.926
MG-MD	-117	-263	29	0.224
MH-MD	-86	-237	64	0.661
MI-MD	-41	-187	105	0.993
MF-ME	-123	-269	23	0.171
**MG-ME**	**-180**	**-326**	**-34**	**0.005**
MH-ME	-149	-300	1	0.053
MI-ME	-104	-250	42	0.374
MG-MF	-57	-203	89	0.944
**MH-MF**	-26	-177	124	1.000
MI-MF	19	-127	165	1.000
MH-MG	31	-120	181	0.999
MI-MG	76	-70	222	0.770

Table S4: Results of the Tukey HSD comparisons between pairs for Flexural strength with difference between the pairs, 95 % confident interval and p-value.

**Flexural strength**				
Sample	*difference*	*95 % confidence interval*		*p adjusted*
MB-MA	**-3.6**	**-6.5**	**-0.7**	**0.005**
**MC-MA**	**-3.1**	**-6.0**	**-0.2**	**0.026**
MD-MA	-2.6	-5.5	0.3	0.112
ME-MA	-2.2	-5.1	0.7	0.308
**MF-MA**	-1.6	-4.5	1.3	0.714
**MG-MA**	**-3.3**	**-6.2**	**-0.4**	**0.013**
**MH-MA**	**-4.6**	**-7.6**	**-1.6**	**0.000**
**MI-MA**	**-3.9**	**-6.8**	**-1.0**	**0.002**
MC-MB	0.5	-2.4	3.4	1.000
MD-MB	1.0	-1.9	3.9	0.976
ME-MB	1.4	-1.5	4.3	0.821
MF-MB	2.0	-0.9	4.9	0.417
MG-MB	0.3	-2.6	3.2	1.000
MH-MB	-1.0	-4.0	2.0	0.977
MI-MB	-0.3	-3.2	2.6	1.000
MD-MC	0.5	-2.4	3.4	1.000
ME-MC	0.9	-2.0	3.8	0.981
MF-MC	1.5	-1.4	4.4	0.763
MG-MC	-0.2	-3.1	2.7	1.000
MH-MC	-1.5	-4.5	1.5	0.811
MI-MC	-0.8	-3.7	2.1	0.994
ME-MD	0.4	-2.5	3.3	1.000
MF-MD	1.0	-1.9	3.9	0.970
MG-MD	-0.7	-3.6	2.2	0.997
MH-MD	-2.0	-5.0	1.0	0.467
MI-MD	-1.3	-4.2	1.6	0.888
MF-ME	0.6	-2.3	3.5	0.999
**MG-ME**	-1.1	-4.0	1.8	0.940
MH-ME	-2.4	-5.4	0.6	0.206
MI-ME	-1.7	-4.6	1.2	0.614
MG-MF	-1.7	-4.6	1.2	0.621
**MH-MF**	**-3.0**	**-6.0**	**0.0**	**0.047**
MI-MF	-2.3	-5.2	0.6	0.231
MH-MG	-1.3	-4.3	1.7	0.907
MI-MG	-0.6	-3.5	2.3	0.999
